# Supplementary material for: Quasi-one-dimensional metallic conduction channels in exotic ferroelectric topological defects
Source: Nat Commun. 2021 Feb 26;12:1306. doi: 10.1038/s41467-021-21521-9 (PMC7910570; doi:10.1038/s41467-021-21521-9)
Supplement: Supplementary file 1 — Supplementary Information [file 41467_2021_21521_MOESM1_ESM.pdf]

Supplementary Information for

Quasi-one-dimensional metallic conduction channels in exotic ferroelectric topological defects

Wenda Yang et al.

Supplementary Note 1. Fabrication of epitaxial $BiFeO_3$ nanoislands

The fabrication procedure for $BiFeO_3$ (BFO) nanoisland arrays in this work is shown in the schematic flowchart of Supplementary Fig. 1a, and this procedure was developed based on a scheme of nano-sphere patterning of the highly epitaxial BFO thin film [1, 2]. In brief, the epitaxial BFO thin film of ~ 40 nm in thickness, along with a ~ 20 nm thick epitaxial $SrRuO_3$ (SRO) layer as bottom electrode, was deposited on the (100)-oriented $SrTiO_3$ substrate by pulsed laser deposition (PLD) using a KrF excimer laser (wavelength λ = 248 nm) with a pulse energy of 300 mJ at 680 °C in oxygen ambient of 15 Pa. Subsequently, the polystyrene spheres (PS) dispersed in a mixture of ethanol and water were transferred onto the as-grown BFO thin film surface to form a closely packed monolayer. The nano-sphere monolayer was then shrunken to the desired size by oxygen plasma to form a discrete/non-intercontacted ordered array.

This procedure was followed by the Ar^+ ion beam etching process for appropriate etching time. After that, the PS nano-spheres were lift-off by chloroformic solution and a periodically ordered BFO nanoisland array was thus obtained, completing the patterning process. Finally, the nanoisland array sample was annealed in oxygen ambiance at temperature of 400 ^oC for 30 min to reduce the defects and residual strain.

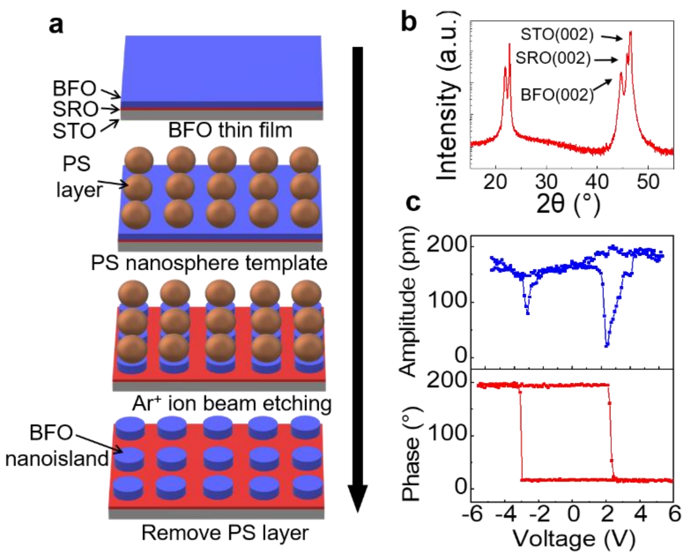

Supplementary Figure 1 Fabrication process of $BiFeO_3$ nanoislands, and their structural and piezoelectric properties. a A schematic flowchart for the fabrication process. **b** The X-ray *θ-2θ* diffraction pattern of the sample. **c** The local piezoresponse hysteresis loops including the butterfly-like amplitude-voltage loop and the phase-voltage hysteresis loop measured onto a randomly selected nanoisland.

Ion beam etching parameters. For the nanoisland fabrication described above, the Ar^+ ion beam etching was conducted in a vacuum pressure of 8.0×10^{-4} Pa at room temperature. During the etching, the incident ion beam was alighted in perpendicular to the sample surface, while the etching parameters were carefully optimized, giving rise to a cathode current of 15.7 A, an anode voltage of 50 V, a plate voltage of 300 V, an ion accelerating voltage of 250 V, a neutralization current of 13 A, and a bias current of 1.2 A.

Supplementary Note 2. Determination of local polarization in nanoislands

A major progress to achieve this work is the reconstruction of domain structure in these nanoislands using the advanced piezoresponse force microscopy (PFM). This PFM allows the simultaneous mapping of vertical (out-of-plane) and lateral (in-plane) amplitudes (abbreviated as V-Amp and L-Amp) and phases (abbreviated as V-Pha and L-Pha). Here, the vertical

polarization orientation can be determined by vertical PFM. In order to determine the local polarization direction (spatial direction), one combines the PFM data recorded at different cantilever orientations (angles), with respect to the sample surface. In most cases, we chose the PFM imaging data at the angles of 0° and 90°, and thus the lateral polarization orientation with respect to the assigned *x*-axis and *y*-axis ([100]-axis and [010]-axis of BFO in our case, respectively), can be determined [3, 4].

Details of the analysis on the two types of domain states: vortex state and center state, are illustrated sequentially in Supplementary Fig. 2. Given the measured PFM data for two selected nanoislands upon the clockwise rotation for 0°, 45°, 90°, and 135° respectively, we can connect the PFM image contrast with the three-dimensional polarization configuration. The different contrasts in the lateral-phase image (L-Pha) represent the two opposite directions of lateral polarization components perpendicular to that of the cantilever. The dark lines in the amplitude image (L-Amp) mark the domain walls where the piezoelectric response should be weak. Based on these PFM images, one can conclude that the lateral polarization components in either of the two nanoislands constitute four quadrant domains. Based on the 0° and 90° measured data (used to determine the polarization components along the [100] and [010]-directions of BFO, respectively) and also given the fact that the lateral polarization components are aligned along the four [110] directions restricted by crystal symmetry, we can conclude that the lateral components must develop the contrasts of four quadrant domains. On the other hand, from the uniform dark contrasts in the vertical PFM images (V-Amp and V-Pha), one can determine that the vertical polarization components are aligned upwards for both nanoislands.

The imaging data for the first nanoisland are presented in Supplementary Fig. 2a. This nanoisland is characterized by four 71° head-to-tail neutral domain walls (71° NDWs) which meet at the core and the directions of vertical polarization components of the four domains are all alighted upwards, forming a flux-closure core region, a typical feature for a quadrant vortex state. The imaging data for the second nanoisland are collected in Supplementary Fig. 2b. This nanoisland accommodates four head-to-head charged domain walls (CDWs) meeting at the core, along with four quadrant domains. These domains all have the upward polarization vertical

components but their lateral components are all aligned towards the core, forming a typical center domain state.

It should be mentioned that such assigned domain structures are also verified by the contrast data at the rotation-angles of 45° and 135° too, validating the domain structure construction. Besides, the C-AFM images of the two nanoislands match well with their domain structure, where the domain walls show different conduction behaviors from the domain interior regions.

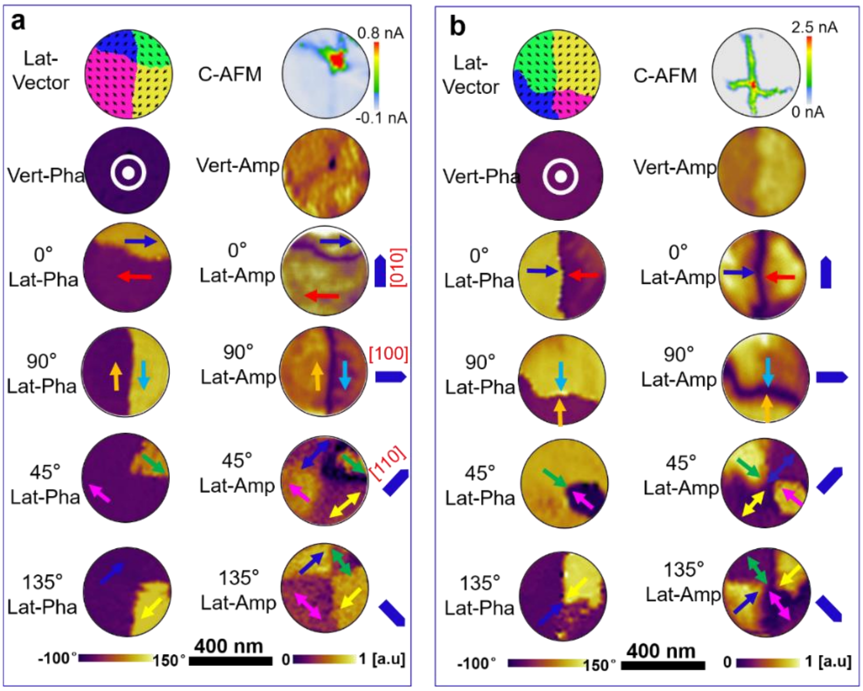

Supplementary Figure 2 Domain structure reconstruction for a vortex domain and a center domain via a conventional method. a,**b** The lateral polarization vector maps and their corresponding C-AFM maps, the vertical PFM phase (V-Pha) and amplitude (V-Amp) images, and the lateral PFM phase (L-Pha) and amplitude (L-Amp) images captured by sample rotation for various angles (0°, 90°, 45°, 135°), for the vortex state (**a**) and center state (**b**). The big blue arrows outside images mark the cantilever direction, and the small arrows inside the images indicate the lateral local orientations of polarization components perpendicular to the cantilever. The double-head arrows illustrate the non-defined directions of local polarization components parallel to the cantilever, given that the marked regions show dark contrast in the lateral amplitude image.

Supplementary Note 3. Determination of polarization distribution via angle resolved PFM

The method described in Section B is the conventional scheme to determine the local polarization direction, while it can't provide more detailed information on local polarization distribution (i.e. both direction and amplitude) in a sufficient accuracy. To provide a comprehensive insight into the nanoscopic polarization distribution, we conducted an angle-resolved PFM for the two topological states, following the method used in earlier literature [5, 6], as shown in Supplementary Fig. 3 & Fig. 4. In this work, the lateral piezoresponse vectors can be determined by combining the lateral PFM image data recorded at different cantilever angles for the same nanoisland.

As shown in Supplementary Fig. 3a, the lateral PFM (amplitude*cos(phase)) images were first recorded by rotating the sample at 9 different angles in sequence. By using the trigonometric curve to fit the angular dependent piezoresponse data of each pixel (position) from the PFM images for the same nanoisland, one is able to determine the amplitude and phase shifts of the sinusoidal function, and then derive the distribution of lateral piezoresponse vectors (with amplitude and phase) for the whole nanoisland. For example, the fitted trigonometric curves of the four selected positions are shown in Supplementary Fig. 3e, from which the direction and amplitude of the piezoresponse vector for a specific location can be derived. After obtaining the piezoresponse vectors for all the pixels in the nanoisland, one can reconstruct the lateral piezoresponse vector map, as shown in Supplementary Fig. 3f.

The derived vector map shows a typical center state and matches well with the C-AFM map and orientation map derived from the previous method. From the new vector map (Supplementary Fig. 3f), one can see no obvious distortion in polarization vector for the four quadrant domains, while we do find some variation in polarization (e.g. partial suppression of amplitude and orientation deviation in the lateral polarization component) in the region adjacent to the center core and CDWs. The variation of polarization in the center core may help suppressing the effect of uncompensated depolarization fields in the core region. This result is also in agreement with the observation for the charge domain walls in Ref. 5 and Ref. 6.

From Supplementary Fig. 4, one can also see the derived vector map for a typical vortex

structure, matching well with the C-AFM map and orientation map derived by the previous method. Further examination of the vectors close to the vortex core reveals that the polarization around the core shows slight rotation and reduced amplitude of the lateral polarization component, which can greatly reduce the possible disclination strain in the core region.

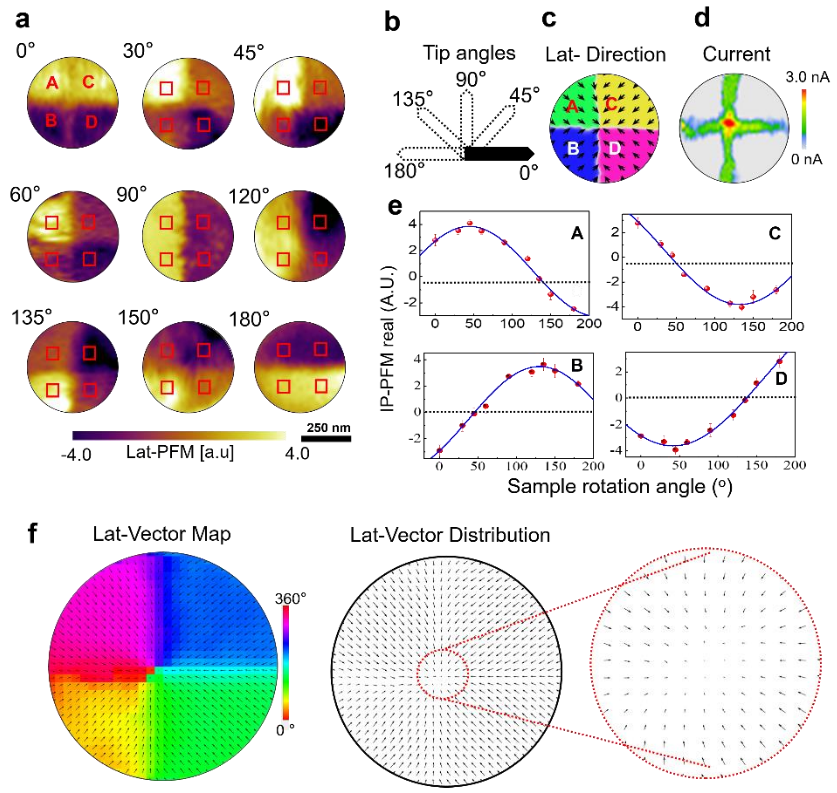

Supplementary Figure 3 The angle-resolved lateral PFM images used to reconstruct the polarization vector map for a selected center domain state. a The lateral PFM images (amplitude*cos(phase)) scanned at 9 different rotation angles for the same nanoisland. **b** A schematic diagram showing the cantilever orientation for each individual scan. **c, d** The simplified lateral direction map (without amplitude) of the nanoisland derived from the conventional method (**c**), and the corresponding C-AFM map (**d**). **e** Examples of the trigonometric fitting curves for the four selected positions using the local piezoresponse data extracted from the Lat-PFM images scanned at 9 different angles, and these data are used to determine both the amplitude and orientation of the lateral local piezoresponse vectors. **f** The reconstructed lateral polarization vector maps for the center state in color contrast and arrow configurations, along with a magnified vector map for the center core region.

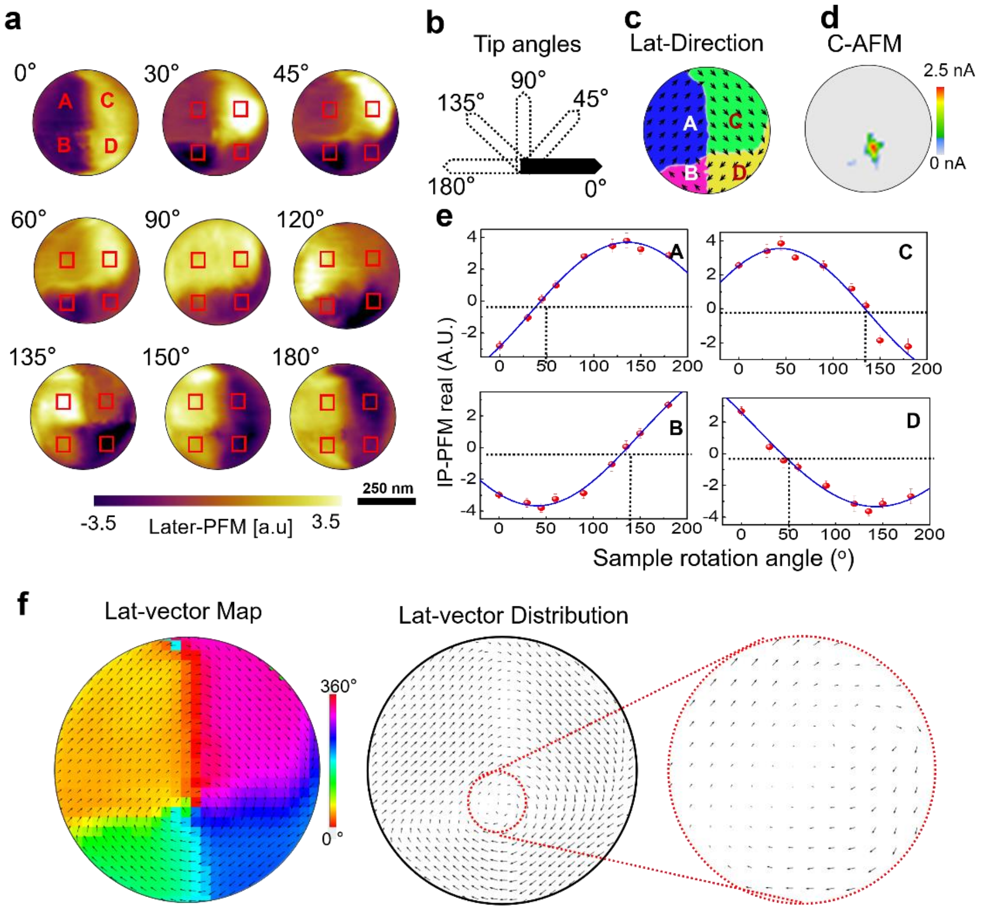

Supplementary Figure 4 The angle-resolved Lateral PFM analysis to reconstruct the polarization vector map for the vortex domain state. **a** The lateral PFM images (amplitude*cos(phase)) scanned at 9 different rotation angles for the same nanoisland. **b** A schematic diagram showing the cantilever orientation for each scan. **c, d** A simplified lateral vector direction map (without amplitude) for the nanoisland derived from the conventional method (**c**), and the corresponding C-AFM map (**d**). **e** Examples of the trigonometric fitting curves for the four selected positions using the local piezoresponse data extracted from the Lat-PFM images scanned at 9 different angles, and these data are used to determine both the amplitude and orientation of the local lateral piezoresponse vector. **f** The reconstructed lateral polarization vector maps for the vortex state in color contrast and arrow configuration, along with a magnified vector map for the vortex core region.

Supplementary Note 4. Creation of topological vortex and center states

Given the domain structure construction whose reliability has been re-confirmed from various methods, it is now possible to implement the creation and removal of the two types of topological domain structures. We start from the initial structure which is a wedge-like pattern (abbreviated as wedge domain state). The vortex and center states can be created by tip-scanning onto a nanoisland under bias voltage, given different writing schemes. For example, a bias of ~ 3.5 V can create a vortex domain state while a bias of ~ 5.5 V allows writing a center domain, as shown sequentially in Supplementary Fig. 5. The schemes also lead to electric modulation of different conduction patterns (in C-AFM map).

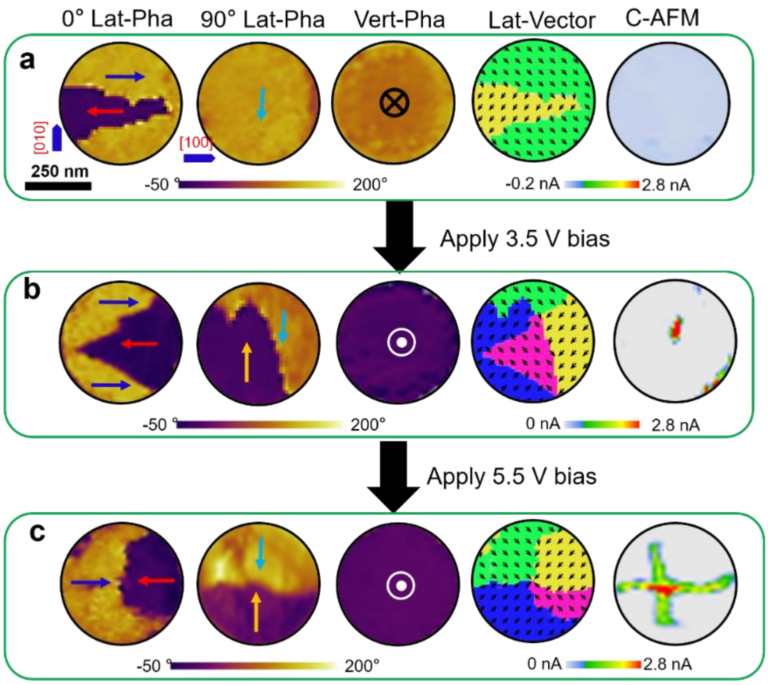

Supplementary Figure 5 The creation of a vortex state and a center state. a-c The domain structures and corresponding C-AFM maps for: the initial wedge domain state with low conduction (**a**), and the vortex state created by a 3.5 V bias scanning (**b**), and the center state obtained after a 5.5 V bias scanning (**c**). The current level for the neutral domain wall is too weak to be visible during imaging in both the initial wedge domain and the written vortex pattern. The micrographs from the left to the right are: the PFM lateral phase images (0° and

90°) used to evaluate the orientation of local polarization component (along the *x*-axis and *y*-axis respectively), the vertical phase image, the lateral local polarization direction map, and the C-AFM map. The small arrows inside the images mark the lateral orientation of the local polarization component perpendicular to the cantilever.

Supplementary Note 5. Conductivity mechanism for the topological cores

To understand the conduction mechanism associated with the two topological states, we tested the temperature (*T*)-dependent conduction for two selected nanoislands: one contains a vortex state and the other a center state (see Supplementary Fig. 6). An immediate glance of the measured current (*I*) - voltage (*V*) data at various temperatures T suggests two distinctly different conductivity regions (see Supplementary Fig. 7): the semiconducting conduction in the low bias range (< 1.7 V) and the metallic-like conduction in the high bias range (> 1.7 V).

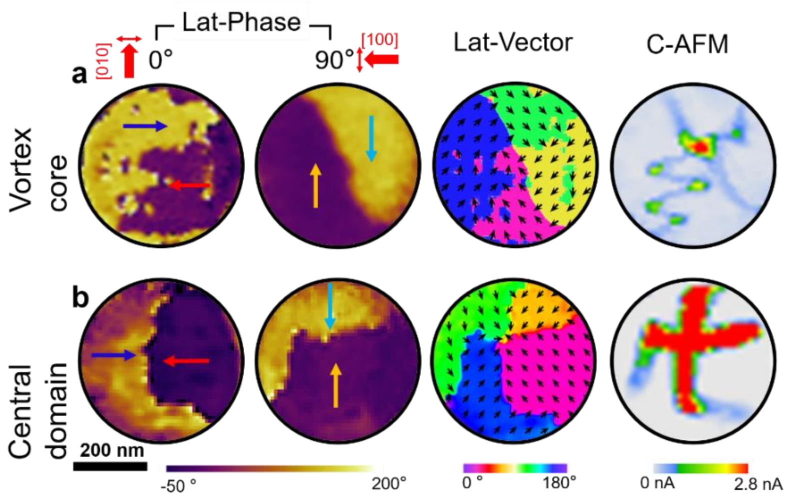

Supplementary Figure 6 The PFM images and corresponding conduction states for the two types of topological states. a The vortex state and **b** center state, which were selected for further temperature-dependent measurements (see Fig. 3 in the main text of the manuscript). The thin arrows inside the images mark the lateral orientations of local polarization components perpendicular to the cantilever.

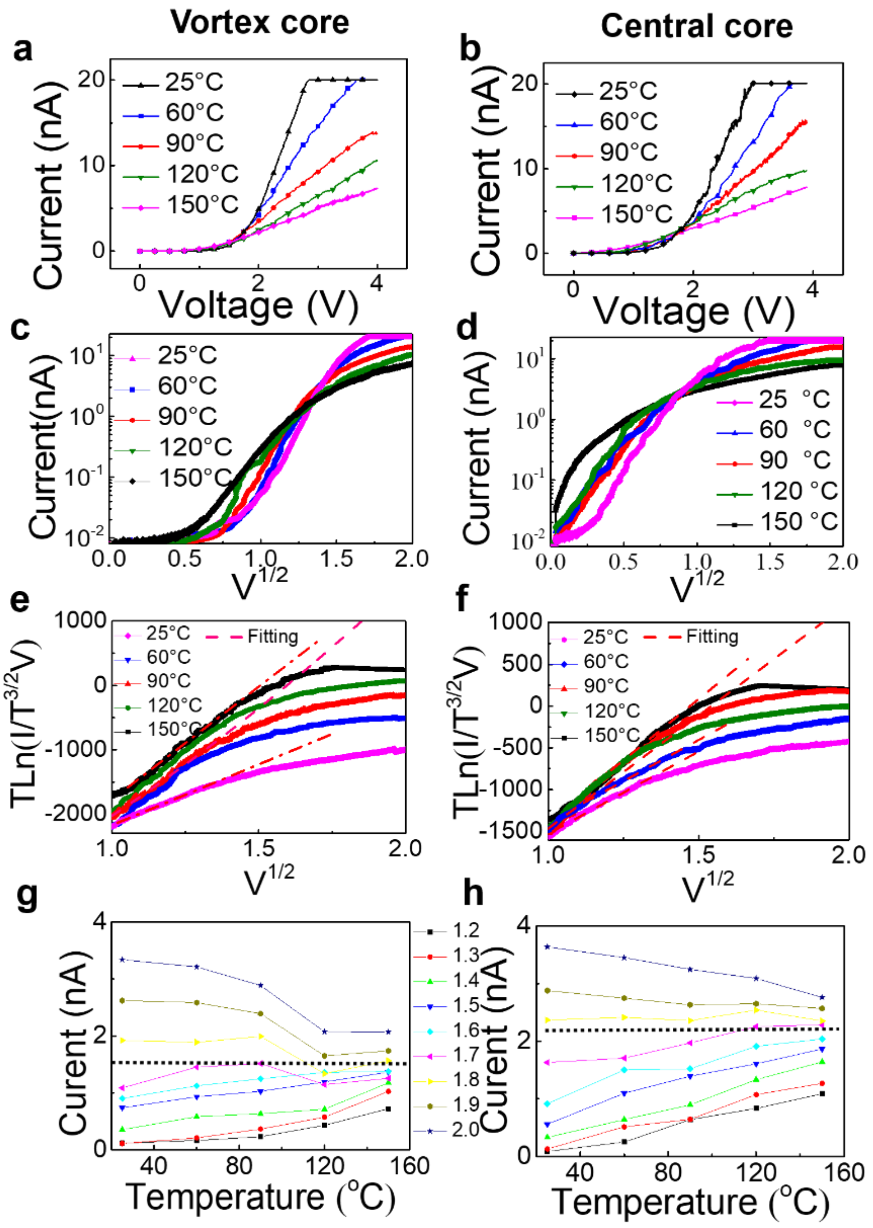

Supplementary Figure 7 The temperature-dependent conduction behaviors for both a vortex core and a center core. a, b The current – bias voltage ($I – V$) curves for the two types of topological cores. **c, d** The temperature dependent I - V curves plotted as ln(I) ~ $V^{1/2}$ to fit the Poole-Frenkel emission model in the low bias range. **e, f** The temperature dependent I - V curves plotted as ln[$I/(T^{3/2}V)$] ~ $V^{1/2}$ to fit the Richardson-Schottky-Simmons emission model in the low bias range. **g, h** The I - T curves for the two types of cores as a function of bias voltage. The I - V curves fit well the Richardson-Schottky-Simmons emission model in the low bias range, while they conform to the metallic conducting mechanism better in the high bias range.

In the low bias range (< 1.7 V), we discuss the measured data and compare them with the signatures of different conduction mechanisms, such as the Fowler-Nordheim mechanism [$\ln(I/V^2) \sim E^{-1}$] [7], the space charge-limited model [$\ln(I) \sim \ln(V)$] [8], the Poole-Frenkel emission model [$\ln(I/V) \sim V^{1/2}$] [9], and the Richardson Schottky Simmons emission model [$\ln(I/(T^{3/2}V)) \sim V^{1/2}$] [10]. Obviously, the data can be fitted well by the Schottky model (see Supplementary Fig. 7e, f) for both the vortex and center cores, indicating that the Richardson-Schottky-Simmons emission is the favored mechanism accounting for the interface-limited conduction.

However, in the high bias range (> 1.7 V), the linear *I-V* curves suggesting more likely the metallic behaviors can be evidenced with the positive temperature coefficient for both the vortex and center cores. Defining the bias threshold as 1.7 V, one finds the metal-like conducting behaviors above the threshold, and the thermionic behaviors [11] below the threshold, for both types of topological cores (see Supplementary Fig. 7g, h).

These observed phenomena in terms of electrical conduction of the topological cores could be explained by the band bending induced by large net polarization - bound charges inside the head-to-head domain cores (Supplementary Fig. 8). The bending leads to a large drop of conduction band below the Fermi level in the cores, thus allowing the attraction of high density of electrons responsible for the metal conduction channels, in analogue to the phenomena observed in charged domain walls [1, 12, 13].

When the metallic channel contacts with electrodes (bottom $SrRuO_3$ electrode and top C-AFM tip), there usually exists a narrow insulating gap and a wedge domain [13] in the contact region close to the electrodes, giving rise to the Schottky barrier between the metallic core and electrode. This narrow gap, if not effectively overcome, would result in semiconducting behavior in the ultra-low bias range. Therefore, it is reasonable that the core region exhibits the thermionic emission behavior in the low bias range. However, in the sufficiently high bias range, the bias overcomes the tunneling barrier from this narrow gap. In this case, the measured resistance comes mainly from the 1D core channel itself. As a result, the metallic conduction becomes dominant over the interfacial thermionic effect. This explanation is consistent with the

observation of a threshold in the *I-V* curve (see Supplementary Fig. 7a, b), below which a nonlinear (non-Ohmic) and semiconducting behavior was identified and beyond which a linear behavior reflecting the metallic behavior was observed

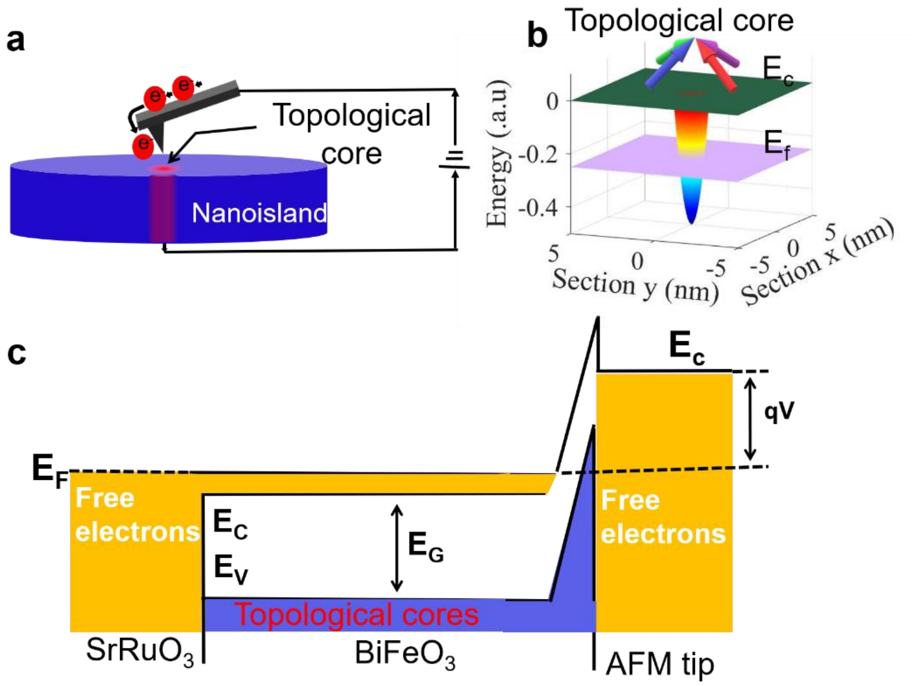

Supplementary Figure 8 A schematic diagram of the band structure in the topological core region. a, b The band bending at the core, creating a conductive channel below the Fermi level. **c** The band structure of the conductive core with electrodes, under an electric field, wherein an insulating gap exists between the conductive channel and AFM tip and this gap can be overcome by a relatively large electric bias. Here, E_F presents the Fermi level, E_G the band gap, and Ec and Ev present the energy levels of conducting band and valence band, respectively.

Supplementary Note 6. Evidences for unstable twisted vortex core

A surprising observation in our experiments is the dynamic behavior of the domain structure, in particular the high sensitivity of the domain wall shape and conduction properties near the core, in response to applied electric bias. One case is the dynamic transition of the

vortex core from a normal vortex state into a twisted state, as driven by an electric bias. Such a twist is basically characterized by the seriously shape-distorted domain wall from roughly straight line, while the twisted state is unstable and a recovery back to the initial state occurs gradually after the bias removal.

Here, it should be mentioned that a high bias (~ 7.0 V) applied to the vortex core is sufficient for the switched distorted core state to survive for 30 min after the bias removal, until the original vortex core state is recovered, as shown in Supplementary Fig. 9. This time interval is sufficient for the PFM probing so that the distorted core state can be captured. Under a smaller bias, the vortex core is only a transient state and its lifetime is insufficient for subsequent PFM imaging. This provides dynamic evidence for the occurrence of dynamically twisted vortex core.

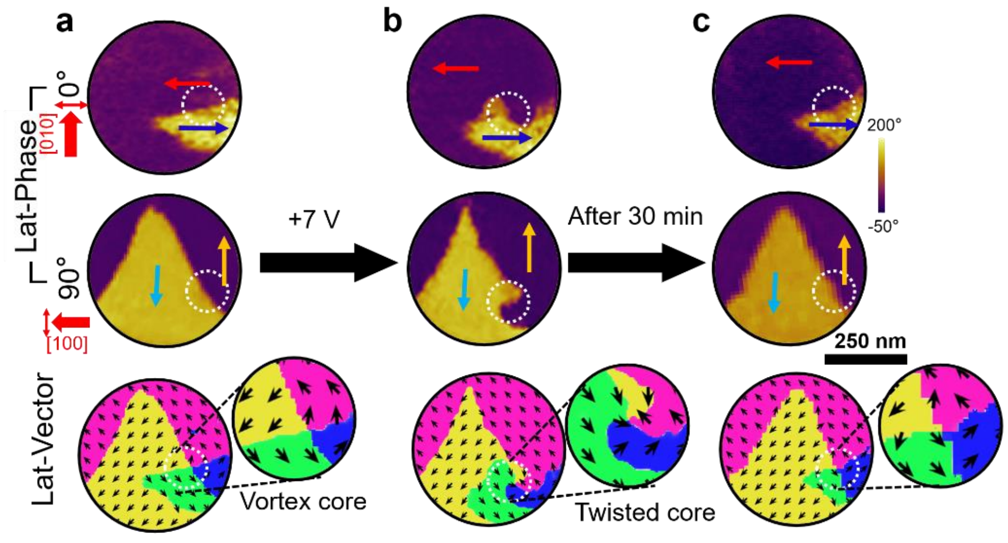

Supplementary Figure 9 The observation of a dynamic twisted vortex core induced by applied electric field. a-c PFM images and polarization vector maps including the initial vortex domain structure (**a**), the center-like twisted core induced by a 7.0 V bias at the vortex core (**b**), though the flux-closure domain pattern remains outside the core region, and the center-like twisted core becomes unstable and returns to the initial vortex core (**c**) at around 30 min after the bias removal. The magnified images for the polarization vector map near the vortex core and twisted core are also presented in the insets beside the vector maps.

To understand the switching dynamics from a vortex core to a twisted core that exhibits high conduction in core region, we conducted the C-AFM probing using various scan rates, as shown in Supplementary Fig. 10. It was revealed that at a very fast scan rate of 78 Hz (the highest scanning rate for our instrument), wherein the dwelling time of the tip at each pixel is as short as 50 μs, the high conduction in the vortex core can still be observable. This implies that the switching from the vortex core to the center like twisted state can be completed within 50 μs. Noteworthily, during the fast scanning, the AFM tip may slant during the high-speed sample movement, such that the contact area between the tip and nanoislands can be enlarged, thus leading to the observed broadening of the high conductive region in the C-AFM images.

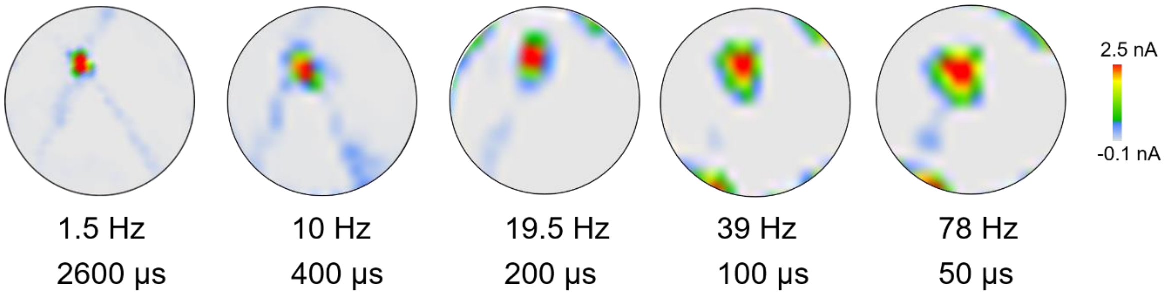

Supplementary Figure 10 The C-AFM maps for a vortex core recorded at different scan rates with a read-out bias of 2.0 V. The scan frequency and corresponding tip dwelling duration at each pixel were also indicated below each individual C-AFM map.

Supplementary Note 7. Lateral sizes of conductive topological cores

Another important issue is the characteristic size of the two types of cores, which is a critical parameter for practical applications and also for understanding. The size can be estimated from the simulated conductive mapping, noting that the simulated results agree well with our observations in terms of the transport properties and dynamic response. Nevertheless, it is impossible to give an experimental estimate directly from the PFM data since the tip used for the C-AFM probing is ~ 30 nm in curvature radius which is too large for probing the

dimension of those ultra-small cores.

The simulated conductivity profiles for the two types of cores are plotted in Supplementary Fig. 11. By measuring the full width at half maxima (FWHM) of line profile of conductivity distribution of the topological cores extracted from the simulated data, one can derive that the core diameters for the two types of topological core are ~ 2.5 nm.

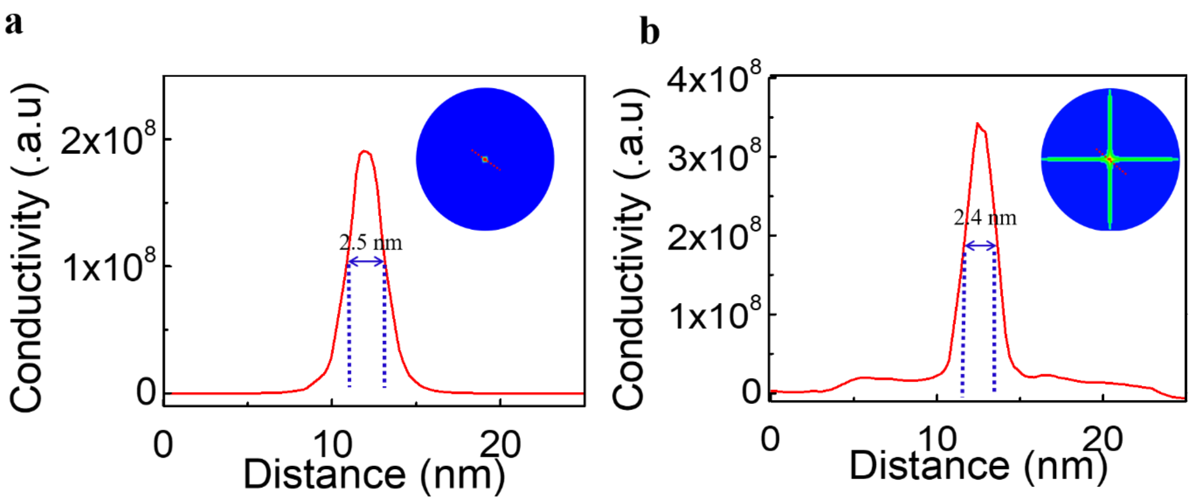

Supplementary Figure 11 The lateral dimensions of the conductive topological cores. a,b The lateral dimensions of the topological cores estimated from the full width at half maxima in the conduction line profiles for the two topological cores, extracted from the simulated conductivity color contours: vortex core (**a**) and center core (**b**). The diameter for each simulated contour is 70 nm.

Supplementary Note 8. Creation and elimination of conductive core states

The detailed information for creation and elimination of the conductive core states can be found in Supplementary Fig. 12 as representative examples. The initial pristine state usually exhibits a wedge-like domain pattern (with downward vertical polarization) of very low conductivity at the walls (abbreviated as wedge domain state). A vortex domain structure can be induced by applying a scanning bias of ~ 3.5 V on the nanoisland, and it will convert to a

center state as the bias is as high as 5.5 V and more. The center domain state can also be switched back (eliminated) to the low conductive wedge domain state by applying a negative bias of -3.5 V. This process can of course be repeatable, and again, the wedge domain state can be switched to vortex state induced by 3.5 V bias, and further applying a bias of 5.5 V leads to the formation of center state.

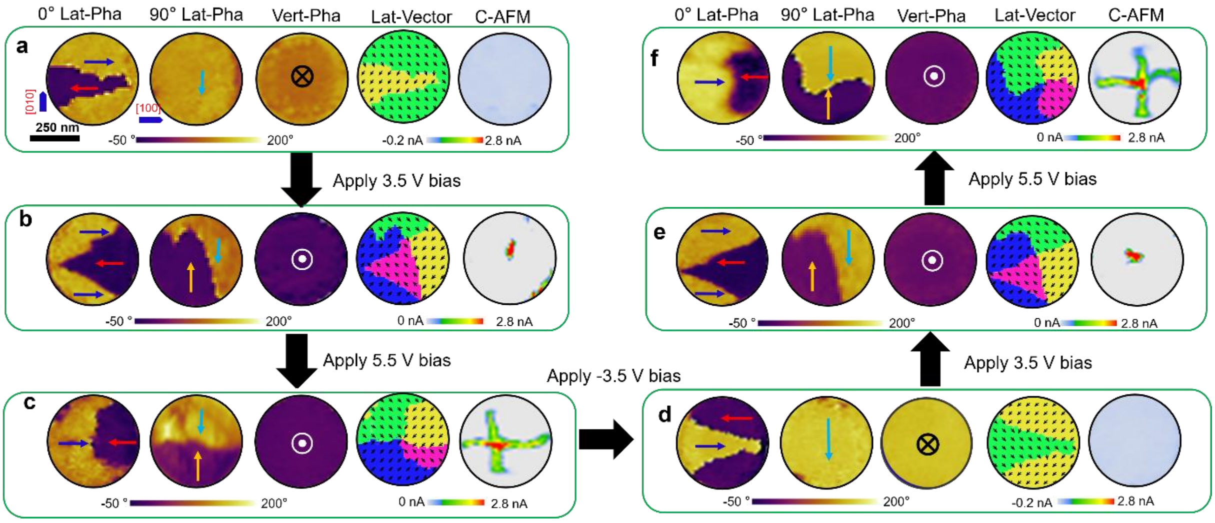

Supplementary Figure 12 The creation and elimination of vortex/center states in a single nanoisland by applying different scanning bias. a-f Biases induced evolution of the domain structures and their corresponding conduction patterns, including: initial low conduction wedge domain state (**a**), creation of a vortex state by applying a bias of 3.5 V (**b**), creation of a center state from a vortex state by a 5.5 V bias (**c**), elimination of a center state (returns to the low conduction wedge domain state) by a -3.5 V bias (**d**), creation of a vortex state by 3.5 V (**e**), and conversion from a vortex state to a center state by 5.5 V (**f**). The micrographs from the left to the right are: the PFM lateral phase images (0° and 90°) used to evaluate the lateral local polarization direction, the vertical phase images, the lateral local vector direction maps, and the C-AFM maps. The panels **a to c** share the same images as Supplementary Fig. 5. The small arrows inside the PFM images present the directions of local polarization component perpendicular to the cantilever.

Selected writing and deleting of the topological states in a nanoisland array were also mapped by the PFM, as shown in Supplementary Fig. 13. It was found that initially all the nanoislands exhibit low conductive wedge domain state, and a tip-scanning under a bias of 5.5 V converts all these nanoislands into the center state each. For better illustration, four nanoislands were circled as shown, and the center states were deleted and converted to the low conduction wedge domain states by the -3.5 V bias writing. Further writing by the 3.5 V bias on two selected nanoislands and 5.5 V bias on the other two nanoislands, created two vortex domain states and two center domain states, respectively. These experiments indicated that both the two types of topological states can be created and eliminated reversibly and individually.

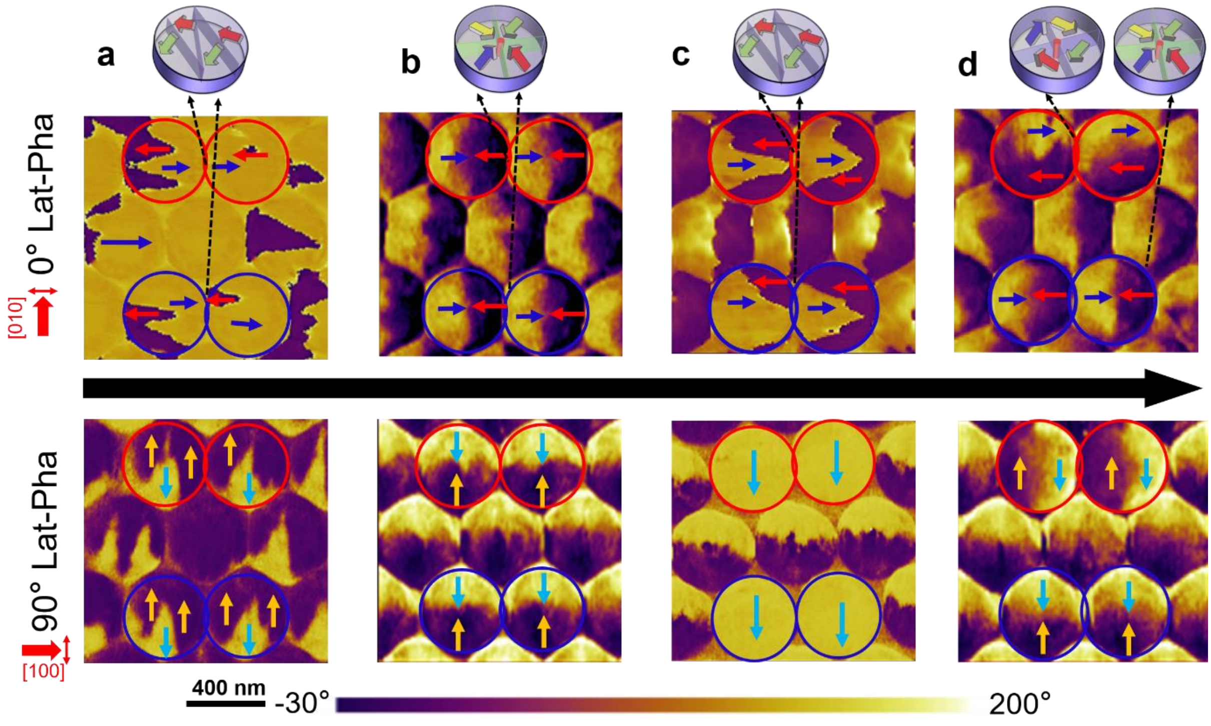

Supplementary Figure 13 The PFM images demonstrating the creation and elimination of the topological states in an array of nanoislands (corresponding to the C-AFM maps of Fig. 5a in the main text). a-d The lateral phase images obtained at sample rotation angles of 0° angle (top row) and 90° angle (bottom row), showing the switching among different topological states: initial low conduction wedge domain states (**a**), center domain states after

applying scanning bias of 5.5 V over the whole array (**b**), elimination of the center domain states (switching back to the wedge domain states) for the four selected nanoislands by applying -3.5 V (**c**), and creation of two vortex states by applying +3.5 V and two center states by applying 5.5 V on the four nanoislands by re-writing (**d**). The four selected nanoislands are marked by the circles. The inset schematics above the PFM images present the different topological states: wedge domain, center and vortex states. The small arrows inside the PFM images present the directions of local polarization component perpendicular to the directions of cantilever.

Supplementary Note 9. Device performances on the topological cores

High temperature retention behaviors. It would be very appreciated if one can test the function of device with top electrodes. However, if the nanoisland is covered by a top electrode, it would become impossible to probe the underneath domain structure. To provide a compromised example, we placed a stationary AFM tip that serves a small top electrode on the topological core, which mimic the structure of a solid-state device.

First, we conducted a retention test for both center and vortex states at a temperature of 150°C for over 120 minutes, and record the time dependent conductive current in topological cores (Supplementary Fig. 14). It was revealed that the conductive levels for both cores are rather stable against retention duration without apparent conduction decline (Supplementary Fig. 14a). This was also verified by the C-AFM maps for both topological states before and after retention test (Supplementary Fig. 14a). The result is much better compared with the written nano-domains in a $Pb(Zr,Ti)O_3$ ferroelectric film reported earlier [14], and the enhanced stability may be attributed to the topological protection and geometric restriction effects.

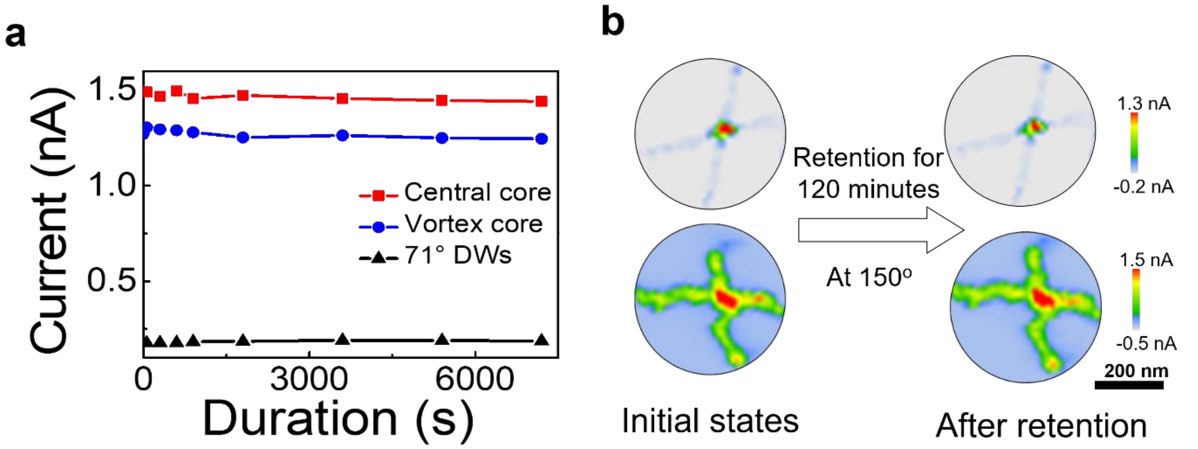

Supplementary Figure 14 The retention behaviors for both a vortex core and a central core at a temperature of 150 °C. a Time dependent conduction current curves for the two topological cores along with a low conductive neutral domain wall tested at a high temperature of 150 °C for over 7200 s. **b** C-AFM maps for both the topological cores before and after the retention test.

Reversible switching properties. Then the switching property on a center core was also tested. It was found that the stationary tip can enable the direct creation and annihilate of the conductive channels in a central core by using electric pulses of ±6.0 V. As shown in Supplementary Fig. 15a, at a read-out bias of 2.0 V, the initial central core exhibits a high conductivity (with a high conduction channel), and it converts to low conduction state (without a high conduction channel) after applying an electric pulse of -6.0 V, while another electric pulse of 6.0 V can recover the high conduction channel again. This process is repeatable for more than 20 cycles, indicating that the high conductive channel can be reversible created and erased by electric pulses. Moreover, the resistance switching behavior also show a good retention at room temperature without apparent decline over four days (see Supplementary Fig. 15b). This indicates the feasibility of using such center core in real memory devices with electrodes.

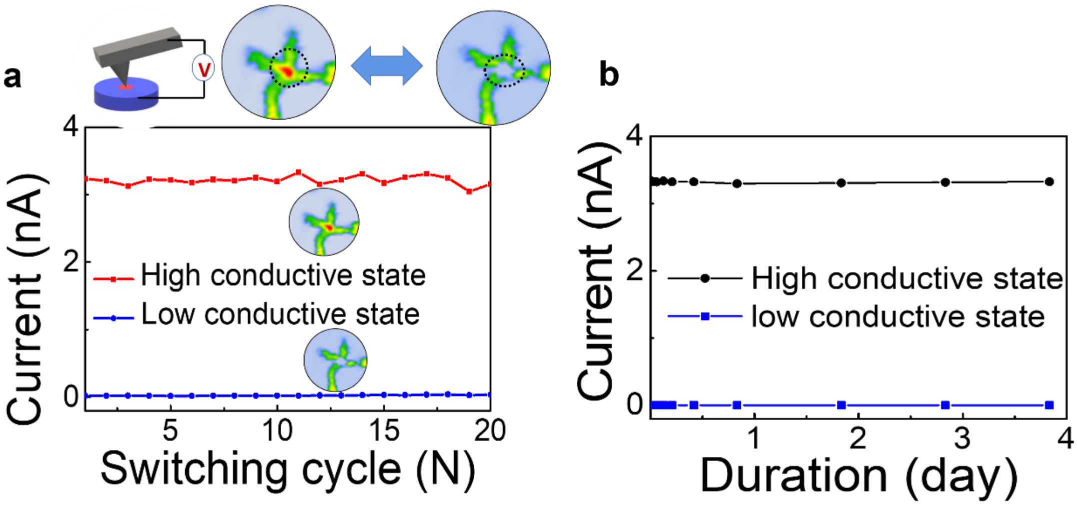

Supplementary Figure 15 Device functions tested by fixing a stationary AFM tip on the center core. a Resistive switching originated from the creation and erasure of the conductive channel in a center cores for more than 20 switching cycles. Insets above **a** illustrate the creating and erasing of conductive channels triggered by electric pulse from the fixed AFM tip on the center core. **b** Retention behaviors for both high and low conductive states at room temperature.

It is also worth of mention that we are not able to reversible switch the vortex core so far. Once the high conduction channel in vortex core is erased, it cannot be recovered back by a reversed pulse. More investigations are needed to improve the switching controllability of vortex core, e.g. searching the reversible switching windows, using larger electrodes.

Fatigue properties. We have also conducted a fatigue test for the center cores (Supplementary Fig. 16). For this, we placed a conductive tip on the topological core and applied electric pulses (with square pulse width of 100 μs, and maximum voltage ±6.0 V) for up to 10^6 switching cycles, and then collected the piezoresponse loops and conductive maps at different intervals, following the method used in earlier literatures [15,16]. For simplicity, we only examined the fatigue behaviors at the core region.

Noteworthily, in a center core, the piezoresponse hysteresis loop (amplitude × cos(phase)) exhibit an 18% decrease in remanent piezoresponse and a doubling in coercive voltage after testing for 10^6 reversal cycles, which can reflect the fatigue behavior of polarization reversal

(Supplementary Fig. 16a, b). After 10^6 fatigue cycles, the center core still exhibits a well-established piezoresponse hysteresis, indicating that it still maintains its good ferroelectricity.

The fatigue behavior of the center core can also be directly reflected by the conductivity decay (Supplementary Fig. 16c). After the fatigue testing for 10^6 switching cycles, the center core can still maintain its ability of reversibly creating and erasing the conductive channels, despite a small loss of conductivity in low resistance state (with a conductive channel). This leads to a small reduction in on/off resistance ratio for ~15%, confirming the good fatigue resistance properties.

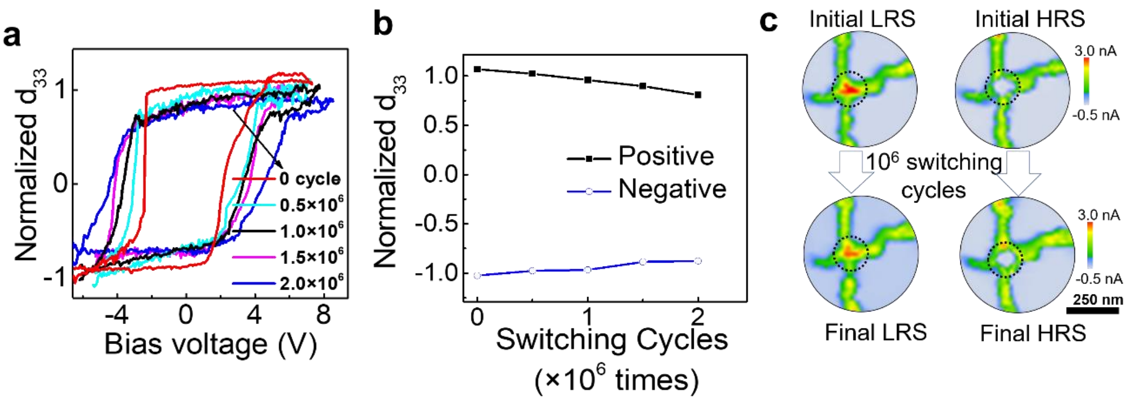

Supplementary Figure 16 The fatigue behaviors for a switchable central core. a The Piezoresponse hysteresis loops of the center core at different switching cycles. **b** Gradual decay in piezoelectric properties against fatigue cycles. **c** C-AFM maps demonstrating the changes in conduction state for both low resistance state (LRS: with a conductive channel) and high resistance state (HRS: without a conductive channel) after 10^6 switching cycles.

References:

1. Tian, G. et al. Manipulation of conductive domain walls in confined ferroelectric nanoislands. *Adv. Funct. Mater* **29**, 1807276 (2019).
2. Tian, G. et al. Observation of exotic domain structures in ferroelectric nanodot arrays

fabricated via a universal nanopatterning approach. *ACS Appl. Mater. Inter* **9**, 37219–37226 (2017).

3. Kalinin, S. et al. Vector Piezoresponse Force Microscopy. *Microsc. Microanal* **12**, 206–220 (2006).
4. Li, Z. et al. High-density array of ferroelectric nanodots with robust and reversibly switchable topological domain states. *Sci. Adv* **3**, e1700919 (2017).
5. Park M, et al. Three-dimensional ferroelectric domain imaging of epitaxial $BiFeO_3$ thin films using angle-resolved piezoresponse force microscopy. *Appl. Phys. Lett.* **97**, 488 (2010).
6. Kim, K. E. et al. Configurable topological textures in strain graded ferroelectric nanoplates. *Nat. Commun.* **9,** 403 (2018).
7. Sze, S. M. *Physics of Semiconductor Devices*, 2nd edn. (Wiley, New York, 1981)
8. Simmons, J. G. & Taylor, G. W. Dielectric relaxation and its effect on the isothermal electrical characteristics of defect insulators, *Phys. Rev. B* **6**, 4793 (1971).
9. Lampert, M. A. & Mark, P. *Current Injection in Solids* (Academic Press, New York, 1970)
10. Scott, J. F. Device Physics of Ferroelectric Thin-Film Memories, *Jpn. J. Appl. Phys* **38**, 2272 (1999).
11. Seidel, J. et al. Conduction at domain walls in oxide multiferroics. *Nat. Mater.* **8,** 229–234 (2009).
12. Crassous, A. et al. Polarization charge as a reconfigurable quasi-dopant in ferroelectric thin films. *Nat. Nanotechnol* **10**, 614–618 (2015).
13. Sluka, T. et al. Free-electron gas at charged domain walls in insulating $BaTiO_3$. *Nat. Commun* **4**, 1808 (2013).
14. Woo J., Hong S., Dong K. M., Shin H., No K. Effect of domain structure on thermal stability of nanoscale ferroelectric domains. *Appl.Phys. Lett.* **80**, 4000-4002 (2002).
15. Colla E. L., Taylor D. V., Tagantsev A. K., Setter N. Discrimination between bulk and interface scenarios for the suppression of the switchable polarization (fatigue) in

$Pb(Zr,Ti)O_3$ thin films capacitors with Pt electrodes. *Appl. Phys. Lett.* **72**, 2478-2480 (1998).

16. Murari N. M., Hong S., Lee H. N., Katiyar R. S. Direct observation of fatigue in epitaxially grown $Pb(Zr,Ti)O_3$ thin films using second harmonic piezoresponse force microscopy. *Appl. Phys. Lett.* **99**, 047601 (2011).
